# Diabetes in an emergency context: the Malian case study

**DOI:** 10.1186/s13031-015-0042-9

**Published:** 2015-05-01

**Authors:** Stéphane Besançon, Ibrahima-Soce Fall, Mathieu Doré, Assa Sidibé, Olivier Hagon, François Chappuis, David Beran

**Affiliations:** Santé Diabète, Mali office, Bamako, Mali; World Health Organization, Mali office, Bamako, Mali; Endocrinology Department, Mali National Hospital, Bamako, Mali; Division of Tropical and Humanitarian Medicine, Geneva University Hospitals, Geneva, Switzerland; Division of Tropical and Humanitarian Medicine Geneva University Hospitals, and University of Geneva, Geneva University Hospitals, Geneva, Switzerland

## Abstract

**Background:**

The World Health Organization proposes 6 building blocks for health systems. These are vulnerable to challenges in many contexts. Findings from a 2004 assessment of the health system in Mali for diabetes care found many barriers were present for the management and care of this condition. Following this assessment different projects to strengthen the healthcare system for people living with diabetes were undertaken by a local NGO, Santé Diabète.

**Case description:**

In March 2012, following a Coup in Bamako, the northern part of Mali was occupied and cut-off from the rest of the country. This had a major impact on the health system throughout the country. Due to the lack of response by humanitarian actors, Santé Diabète in close collaboration with other local stakeholders developed a humanitarian response for patients with diabetes. This response included evacuation of children with Type 1 diabetes from northern regions to Bamako; supplies of medicines and tools for management of diabetes; and support to people with diabetes who moved from the north to the south of the country.

**Discussion:**

It has been argued that diabetes is a good tracer for health systems and based on Santé Diabète’s experience in Mali, diabetes could also be used as a tracer in the context of emergencies. One lesson from this experience is that although people with diabetes should be included as a vulnerable part of the population they are not considered as such. Also within a complex emergency different “diabetes populations” may exist with different needs requiring tailored responses, such as internally displaced people versus those still in conflict areas. From Santé Diabète’s perspective, the challenge was changing the ways it operated from a development NGO to an emergency NGO. In this role it could rely on its knowledge of the local situation and its function as part of the post-conflict situation.

**Conclusion:**

The lessons learnt from this experience by Santé Diabète in Mali may be useful for other NGOs and the humanitarian response in general in addressing the challenge of managing non communicable diseases and diabetes in conflict and disaster situations in countries with weak health systems.

## Background

The World Health Organization (WHO) states that the health system is comprised of all the “activities whose primary purpose is to promote, restore and maintain health” [1]. Health systems are intimately linked to the other socio-political elements in a given country and therefore different models of health systems exist [2]. However, each health system can be described based on the six key functions they must deliver [2]: (1) Service delivery; (2) Healthcare workforce; (3) Information; (4) Medical products, vaccines and technologies; (5) Financing; and (6) Leadership and governance.

In caring for people with diabetes the health system needs to prevent complications in addition to any other negative physical and psychosocial impacts of this condition [3,4]. However, health systems in Low Income Countries (LIC) have been found to be weak at delivering the services that their populations need as they are not tailored to care for people with chronic diseases [5,6]. Failings in one or many of these 6 elements can be the cause of this. Based on this, a Rapid Assessment Protocol (RAP) to assess the barriers to diabetes care in LICs was developed by the International Insulin Foundation (IIF: UK registered charity active in the area of health systems and access to diabetes care) with the aim to assess the barriers to access to diabetes care and medicines [7]. This approach allows for an overall vision of the 6 elements of the health system and where these may fail in delivering appropriate diabetes care.

In 2004 the IIF in partnership with Santé Diabète (French NGO active in Francophone West Africa in the area of diabetes) carried out this RAP in Mali. Mali was chosen for this study as like many other countries in sub-Saharan Africa it faced an increasing burden of diabetes due to increases in urbanization, the challenges of the nutritional transition and increasing sedentary behavior. The Malian health system is also confronted by many challenges with regards to the 6 WHO building blocks and it was of interest to the IIF, Santé Diabète and the Malian Ministry of Health to see how these impacted the management of diabetes. Finally having Santé Diabète active in Mali and collaborating closely with local stakeholders and institutions meant that the recommendations from the RAP could be implemented.

Mali is located in West Africa with a population of 14.8 million people and a life expectancy at birth of 57 years for men and women [8]. Total expenditure on health per capita per year is Int.$ 74 representing 5.8% of total Gross Domestic Product. It is estimated that 31% of total deaths are due to Noncommunicable diseases (NCD), with 2% due to diabetes [8]. The International Diabetes Federation (IDF) estimates the prevalence of diabetes in Mali at 1.28% of the adult population [9], but local experts would state that this seriously underestimates the true burden. The prevalence of Type 2 diabetes in the African Region is estimated to be 4.9% (20–79-year olds) [10].

The findings from the 2004 study found many barriers with regards to the 6 building blocks of the health system. For example, in terms of service delivery, there were no guidelines or treatment protocols, no organization and coordination of care and referrals, sub-optimal use of the existing health pyramid in Mali, long waiting times and a lack of patient education [11]. An absence of care outside of Bamako (the capital city) meant that many people from all of Mali had to travel to Bamako for care. Specialists were only present in Bamako and there was a clear lack of human resources in terms of overall number and training. At the time of the assessment there were only 2 specialists (one diabetologist and one endocrinologist) practicing in all of Mali. In terms of data and information there was no standardized means of collecting information on patients and there was an overall lack of data on the number of people with diabetes in Mali. Serious problems were present in terms of the availability and affordability of medicines and other supplies. Insulin was not widely available and cost approximately US$ 11 per vial. Syringes and diagnostic tests were also not readily available and posed a financial burden to those who needed to access them. Issues with the supply chain for both medicines and laboratory equipment were identified. The patient and their family had to bear the entirety of the financial burden of diabetes as no government or other support was available. Leadership and governance were lacking with no government policy or strategy addressing diabetes and no organizational structure at a national level coordinating the government’s response to diabetes or NCDs.

This resulted in the life expectancy of a child with Type 1 diabetes being 1 year after diagnosis, in comparison to normal life expectancy in the “Western world”. On average per month a patient in Bamako spent $21.24 on diabetes care (assuming 1 blood glucose measurement per month, 8 syringes per month, 1 vial of insulin at an average cost of $10.88 in the public sector, 1 monthly consultation, and travel costs) representing nearly 70% of per capita GDP [12]. In contrast in Mozambique and Zambia other countries in sub-Saharan Africa where this research was carried out monthly costs were equivalent to US$ 22.8 and US$ 16.59 respectively [13].

These results were then used by Santé Diabète, in collaboration with the Ministry of Health, local specialists and the WHO Country Office to develop a comprehensive strategy to address the barriers highlighted above. The overall project included training of diabetes “referral” doctors throughout the country, training for other health professionals in the area of diabetes and the creation of specialized university diplomas in the area of diabetes. In addition improvements were made with the availability of diagnostic tools and decreasing the price of medicines. Diabetes registers were created, as were different elements to improve service delivery such as development of specialized and decentralized diabetes consultations, strengthening of patient education and targeting specific complications. These projects were destabilized due to the crisis that started in Mali in March 2012.

## Case description

On the 22^nd^ of March 2012, Mali was shaken by a coup carried out by part of the Malian armed forces [14]. In the beginning of April 2012 the main towns of the north of Mali, Gao, Kidal and Timbuktu were overtaken by Touareg rebels seeking independence from Bamako [15]. In July the same year Islamists took control of the whole of northern Mali and in January 2013 these forces launched an offensive to take over the south [16,17]. The French government intervened militarily in its former colony to stop the progression of these militants. This destabilization of the country, its government and health system impacted the 6 building blocks of the health system. In the north of Mali this had a direct impact whereas in the south the impact was more indirect due to people exerting more pressure on the already weak health system as they fled the conflict zone. The impact of this situation on the 6 building blocks of the WHO in terms of diabetes in the two areas of Mali is summarized in Table 1. Because of this people with diabetes faced a whole series of challenges in terms of access to care and medicines [18].

**Table 1:** **Impact of conflict on the health system in the north and south of Mali** [18]

**WHO Health System Building Block**	**North of Mali**	**South of Mali**
Service delivery	• Limited services provided by NGOs	• Extra burden on existing services due to the number of internally displaced population
	• Only 7.9% of health facilities were providing diabetes services	• Limited availability of free health services
	• Services provided by UN agencies or NGOs versus Malian health service	
	• 65% of health facilities were not functional	
Healthcare workforce	• Flight from conflict zone	• Insufficient health workers to accommodate the massive internal displacement (more than 300,000 internally displaced persons mainly to the region of Mopti)
	• Limited number of NGOs and local resident health workers providing limited services	
Information	• Lack of accurate and credible health information	• Lack of capacity to collect regular data in addition to supplementary data to manage crisis
	• Malaria and measles epidemics detected by NGOs	
Medical products, vaccines and technologies	• Complete interruption of supplies	• Lack of supplies at facilities for the people with diabetes already being managed
	• Destruction of existing infrastructure	
		• Additional burden further strained existing limited resources
Financing	• Role of United Nations and NGOs versus government	• Health services partly financed by government and bilateral and multilateral donors, UN agencies and NGOs
		• The population in the south received no additional support and due to the influx of people from the north and crisis situation actually received less support than prior to the crisis
	• Limited partners support (44% of the health financing in Timbuktu Region)	
Leadership and governance	• Absence of local Malian government authority in north of country	• Instability of the transitional government
		• Lack of leadership for policy issues

As of April 2012 a massive humanitarian response was organized to help the 2.24 million people living in areas directly impacted by the conflict and 198,500 displaced people [19,20]. To address this situation two parallel responses were developed, one by the Ministry of Health’s technical staff and the other developed by humanitarian actors. Both of these were then combined into a Common Humanitarian Action Plan. The overall resources allocated to this crisis were US$ 150 million for the overall humanitarian response and US$ 9.5 million specifically for health [19,20].

In asking for support for people with diabetes Santé Diabète received many negative responses from traditional donors. For example the Office for the Coordination of Humanitarian Affairs (OCHA) and United Nations Children's Fund (UNICEF) declined to support diabetes related activities despite the small amount requested, US$ 41,000 from OCHA and US$ 12,700 from UNICEF. The response from OCHA was “diabetes is not in the framework and not a emergency” and UNICEF stated “Diabetes is not a priority” [21]. It was not the actual proposals developed by Santé Diabète that were rejected, but the population in critical need of diabetes services not seen as a priority by those responding to the crisis. The reason for this is that people with NCDs and diabetes were not included in the “priority list”, as they were not considered as vulnerable. Diabetes should be seen as a priority as people with this condition are vulnerable even in normal circumstances, a fact being exacerbated during a period of crisis.

Santé Diabète decided to propose an emergency strategy for people with diabetes building off its knowledge and experience as a development NGO active in Mali since 2001. This strategy focused on two target populations:

1. People with diabetes living in the north who were still in that area
2. Internally displaced people with diabetes

Santé Diabète also debated its response with regards to Malian refugees with diabetes in neighboring countries and initiated discussions with the United Nations High Commissioner for Refugees (UNHCR). However, due to limited resources Santé Diabète was unable to respond to the needs of this group.

Given this lack of response from major donors as well as actors on the ground, Santé Diabète was still able to find funding from different sources. The partners who responded positively were the French Crisis Center and the French Development Agency, the Rhône Alpes region in France and private donations. In addition the private sector contributed some medicines. With the technical support of the WHO Country Office the diabetes emergency response was launched in partnership with the Ministry of Health, the Department of Endocrinology and Diabetology of the “Hôpital du Mali” and the National Federation of Malian Diabetics.

Because of the technical and financial constraints, Santé Diabète decided to focus on 4 of the 6 health system blocks. The first and fundamental block was access to medicines. Santé Diabète was able to supply essential diabetes medicines and insulin to the north of the country by using diabetes patient associations, health professionals and humanitarian NGOs active in the north of the country. Santé Diabète supported 1,814 people in the north of Mali with medicines. Testing equipment was also supplied to areas where this equipment had been destroyed or was no longer available, and Santé Diabète created and supplied different kits for the management of diabetic comas and diabetic foot complications (See Table 2). Malian specialists created these kits and Santé Diabète provided the logistical support for this.

**Table 2:** **Specialized diabetes kits developed by Santé Diabète**

**Tools included in specialized diabetes kits and average quantities needed per patient**			
**Foot kit**		**Diabetic coma**	
Antibiotic	Quantity of each one for 7 days per patient	Insulin syringes U-100	1 box of 100
3 families (Fluoroquinolone; Macrolides, lincosamides and streptogramines; Beta-lactam)		Rapid acting insulin	1 vial of 10 ml
		Blood Glucose Meter	1
Metronidazole	21 bottles 100 ml	Glucose strips	1 box of 50
Dakin’s solution	1 bottle of 250 ml	Urine test strips	1 box of 50
Compresses (40*40 cm)	2 boxes of 10	Lactated Ringer’s solution	20 bottles 500 ml
Bandages 10 x 4.5 cm B/12	1 box of 12	Saline solution	15 bottles 500 ml
		Urinary catheter	2
		Urinary catheter bag	2
		Gastric catheter	2
		Quinine	12 vials of 400 mg

These kits were then supplied to those in need via local authorities from Timbuktu, humanitarian NGOs and diabetes associations. The emergency kits prepared by Santé Diabète enabled to care of 32 people with diabetic foot complications and 15 people in diabetic coma. These actors also assisted in data collection with a simple data collection sheet developed by Santé Diabète. This was used to assess the needs for the kits and medicines as well as identify people requiring support. Using this information helped Santé Diabète identify patients from the north who had moved to the south. The “service delivery” element was also seen as important and Santé Diabète provided technical support to humanitarian NGOs active in the north of Mali and health professionals still in these regions via telephone support with specialists at the “Hôpital du Mali” for complex cases.

Service delivery was also reinforced in the south of the country where there was a large influx of displaced people. One particular element of service delivery that was addressed was the evacuation of children with Type 1 diabetes from the north of Mali to Bamako. Due to the complexity of the management of this condition known children with this condition were brought to Bamako as soon as the crisis started. Santé Diabète also supplied materials and medicines to facilities in the south to help them cope with the increased patient load. Although different NGOs and the International Organization for Migration (IOM) had within their remit to care for internally displaced people they asked Santé Diabète to manage people with diabetes. Santé Diabète was therefore responsible for the care and medicines of 150 people with diabetes. Finally one can argue that although limited, Santé Diabète through its activities was able to contribute to the “Financing” element of the emergency response. Santé Diabète’s response is summarized in Table 3.

**Table 3:** **Santé Diabète’s response to the crisis using the WHO’s 6 building blocks**

**WHO Health System Building Block**	**Santé Diabète’s response**
Service delivery	• Support to other NGOs and doctors active in the north of Mali
	• Support to facilities in the south to cope with additional burden
	• Transporting children to Bamako
Information	• Development of simple tools to collect information on people with diabetes
	• Use of existing networks for information on the situation
Medical products, vaccines and technologies	• Donations
	• Development of special kits
Financing	• Covering all costs for those in need within its limited means

The health system elements of workforce and leadership and governance were not addressed. Santé Diabète neither had the resources nor considered as its mandate to provide medical personnel specifically dedicated to diabetes. However, as described in the response, support to services were provided through other organizations on the ground. The contacts of Santé Diabète with OCHA, UNICEF, WHO and IOM should be considered as an attempt to respond to the needs of people with diabetes from a service delivery rather than a leadership and governance perspective.”

## Discussion

As diabetes is considered a good tracer for health systems [5,22,23], this case study might provide useful insight into the humanitarian response for NCDs and diabetes. The lessons learnt from Santé Diabète’s experience in managing diabetes in an emergency context are relevant to the different elements of the 6 building blocks. Although these building blocks are usually utilized for stable health systems they remain relevant in times of emergency as people still need to access health care provided by a system be it delivered by local facilities, NGOs or a UN agency.

This case study highlights the lack of leadership and governance in including NCDs and diabetes on the agenda of humanitarian actors. In September 2011 the United Nations held a Summit on NCDs, its second health-related summit after its 2001 meeting on HIV/AIDS. Despite being the main cause of mortality worldwide with 63% of total deaths [24], NCDs have not been firmly placed on the development agenda [25,26]. Although often thought of as “diseases of the rich” close to 80% of NCD deaths occur in Low and Middle-Income Countries. Following this the WHO has developed a Global Action Plan for NCDs that include the following statements regarding NCDs and the humanitarian response [27]:

- “It must be ensured that the use of these services does not expose the users to financial hardship, including in cases of ensuring the continuity of care in the aftermath of emergencies and disasters.”
- “Improve the availability of life-saving technologies and essential medicines for managing NCDs in the initial phase of emergency response.”

Despite clear recommendations from the WHO, prioritizing these diseases both on global level and also specifically for emergencies, other UN organizations and humanitarian actors have not followed this guidance. As highlighted by Demaio et al. [28] NCDs have not received the attention necessary in emergency contexts. In Mali the WHO in its role of coordinator of the health cluster was the only international actor trying to address the NCD issue. This was reinforced by a change of leadership in WHO in Mali which allowed awareness to be raised about the need to take NCDs into account among the priority health issues during the Malian crisis.

With regards to information, medical products, service delivery, human resources and financing Spiegel et al. [29] provide guidance in terms of what should be done in humanitarian crises. This includes registration of people with chronic conditions that can be cared for locally, including diabetes and cardiovascular disease. As done by Santé Diabète, Spiegel et al. [29] propose the creation of treatment kits for health facilities as well as simplified care guidelines for the use at home. Regarding human resources the recommendation is that chronic disease care should be included in the training of humanitarian workers. Finally, the issue of cost of management of diabetes and NCDs and how the humanitarian response needs to adequately plan for this.

This case study also raises the issue of vulnerability. Vulnerable populations are more prone to face the adverse effects of a humanitarian crisis [30] and people with diabetes clearly fit into this category. This specific population can be seen as vulnerable due to their continuous need for health care and medicines and the financial burden this may place on them. Type 1 diabetes affects children, already a category of vulnerability on its own. The needs of people with diabetes are already complex and the added strain of a crisis situation means that this sub-population, as all populations with chronic diseases, need special attention [31].

In an emergency setting there is also not one single “diabetes population”, but several each with specific needs. These sub-populations included people still in the conflict area, internally displaced people, refugees and also the population in the south of Mali. All these populations contain individuals with diabetes, but the response to each set of needs may be very different. For Malian refugees these were people who may have lost everything and had to flee their homes for neighboring countries, versus people internally displaced having lost their home, but being able to stay with family and friends in Bamako and still being in their country. In the north the health system was completely destroyed, versus in the south due to the crisis the health system became overburdened. All these sub-populations need to be addressed in different ways with a variety of actors involved.

Santé Diabète in its response to 3 out of 4 of these populations had to shift its focus from being a development NGO to managing development projects in a humanitarian crisis as well as developing a response to the emergency situation in Mali. Its response could rely on its vast knowledge and experience of the Malian context and links with local partners to address challenges it faced on the ground. Long-term experience of a country is something that traditional humanitarian organizations do not have due to their modus operandi and short-term focus in a given setting. In addition, having carried out a situation analysis of the health system in 2004 allowed Santé Diabète an in-depth knowledge of the Malian health system. It has been proposed that humanitarian organizations in their assessment of needs and response include NCDs [30]. Santé Diabète through its existing projects and partnerships is also actively involved in the post-emergency reconstruction of the health system. By intervening in the crisis situation it has guaranteed continuity of care with regards to medicines, service delivery, information and financing.

## Conclusion

The lessons from this case study may be applicable to other LICs where frequent humanitarian emergencies place strain on already weak health systems. These health systems do not provide appropriate management for NCDs including diabetes meaning that in a time of humanitarian emergencies this already vulnerable population faces additional challenges. The lack of priority given to this group of individuals in humanitarian emergencies needs to be addressed as well as how the response for chronic conditions needs to build on existing local expertise to benefit the emergency response, but also plan for the post-emergency reconstruction.

## References

[1] WHO (2000). The World Health Report 2000 - Health systems: improving performance.

[2] WHO (2007). Strengthening health systems to improve health outcomes: WHO’s framework for action.

[3] Clark NM (2003). Management of chronic disease by patients. Annu Rev Public Health.

[4] Travis P, Bennett S, Haines A, Pang T, Bhutta Z, Hyder A (2004). Overcoming health-systems constraints to achieve the Millennium Development Goals. Lancet.

[5] Beran D (2012). Health systems and the management of chronic diseases: lessons from Type 1 diabetes. Diabetes Management.

[6] Mills A (2014). Health care systems in low- and middle-income countries. N Engl J Med.

[7] Beran D, Yudkin J, de Courten M (2005). Assessing Health Systems for Insulin-Requiring Diabetes in sub-Saharan Africa: Developing a 'Rapid Assessment Protocol for Insulin Access'.

[8] Country Health Profiles. [http://www.who.int/countries/]

[9] IDF (2013). International Diabetes Federation Diabetes Atlas.

[10] Peer N, Kengne AP, Motala AA, Mbanya JC (2014). Diabetes in the Africa Region: an update. Diabetes Res Clin Pract.

[11] International Insulin Foundation, Santé Diabète (2004). Final Report of the International Insulin Foundation on the Rapid Assessment Protocol for Insulin Access in Mali.

[12] International Insulin Foundation (2005). Diabetes Foundation Report on insulin-requiring diabetes in sub-Saharan Africa.

[13] Beran D, Yudkin JS (2010). Looking beyond the issue of access to insulin: what is needed for proper diabetes care in resource poor settings. Diabetes Res Clin Pract.

[14] Nossiter A (2012). Soldiers Overthrow Mali Government in Setback for Democracy in Africa.

[15] Hirsch A (2012). Mali rebels declare independence in north as fears grow over extremist links.

[16] Mali: Islamist Armed Groups Spread Fear in North. [http://www.hrw.org/news/2012/09/25/mali-islamist-armed-groups-spread-fear-north]

[17] Hirsch A, Willsher K (2013). Mali conflict: France has opened gates of hell, say rebels.

[18] WHO Mali Country Office (2013). Implementation report of health interventions in response to the humanitarian crisis in Mali.

[19] Appel global pour le Mali 2012. [http://www.unocha.org/cap/appeals/appel-global-pour-le-mali-2012]

[20] Mali Tableau de Bord Humanitaire. [http://www.unocha.org/mali/infographies/tableaux-de-bord-humanitaires]

[21] Besancon S, Traore SA (2013). [Diabetes, a public health challenge for Mali]. Soins.

[22] Kessner DM, Carolyn EK, Singer J (1973). Assessing health quality: the case for tracers. N Engl J Med.

[23] Nolte E, Bain C, McKee M (2006). Diabetes as a tracer condition in international benchmarking of health systems. Diabetes Care.

[24] WHO (2010). Global status report on noncommunicable diseases.

[25] Nugent R, Feigl A (2011). Where Have All the Donors Gone? Scarce Donor Funding for Non-Communicable Diseases.

[26] Stuckler D, King L, Robinson H, McKee M (2008). WHO's budgetary allocations and burden of disease: a comparative analysis. Lancet.

[27] WHO (2013). Global Action Plan for the Prevention and Control of Noncommunicable Diseases 2013–2020 - Revised draft (Version dated 11 February 2013).

[28] Demaio A, Jamieson J, Horn R, de Courten M, Tellier S (2013). Non-communicable diseases in emergencies: a call to action. PLoS Curr.

[29] Spiegel PB, Checchi F, Colombo S, Paik E (2010). Health-care needs of people affected by conflict: future trends and changing frameworks. Lancet.

[30] UNISDR, WMO: Disaster Risk and Resilience. In*.*; 2012.

[31] Beran D (2014). Developing a hierarchy of needs for Type 1 diabetes. Diabet Med.

